# LED omics in Rocket Salad (*Diplotaxis tenuifolia*): Comparative Analysis in Different Light-Emitting Diode (LED) Spectrum and Energy Consumption

**DOI:** 10.3390/plants12061203

**Published:** 2023-03-07

**Authors:** Aphrodite Tsaballa, Aliki Xanthopoulou, Ilektra Sperdouli, Filippos Bantis, Anastasia Boutsika, Ioanna Chatzigeorgiou, Eleni Tsaliki, Athanasios Koukounaras, Georgios K. Ntinas, Ioannis Ganopoulos

**Affiliations:** 1 Institute of Plant Breeding and Genetic Resources, Hellenic Agricultural Organization DIMITRA (ELGO-Dimitra), GR-57001 Thermi, Greece; 2 Department of Horticulture, School of Agriculture, Aristotle University of Thessaloniki, GR-54124 Thessaloniki, Greece

**Keywords:** LED lights, energy use efficiency, water use efficiency, transcriptomics, photosynthesis, controlled environment room

## Abstract

By applying three different LED light treatments, designated as blue (B), red (R)/blue (B), red (R) and white (W) light, as well as the control, the effect on *Diplotaxis tenuifolia* phenotype (yield and quality), and physiological, biochemical, and molecular status, as well as growing system resource use efficiency, was examined. We observed that basic leaf characteristics, such as leaf area, leaf number, relative chlorophyll content, as well as root characteristics, such as total root length and root architecture, remained unaffected by different LEDs. Yield expressed in fresh weight was slightly lower in LED lights than in the control (1113 g m^−2^), with R light producing the least (679 g m^−2^). However, total soluble solids were significantly affected (highest, 5.5° Brix, in R light) and FRAP was improved in all LED lights (highest, 191.8 μg/g FW, in B) in comparison to the control, while the nitrate content was less (lowest, 949.2 μg/g FW, in R). Differential gene expression showed that B LED light affected more genes in comparison to R and R/B lights. Although total phenolic content was improved under all LED lights (highest, 1.05 mg/g FW, in R/B), we did not detect a significant amount of DEGs in the phenylpropanoid pathway. R light positively impacts the expression of the genes encoding for photosynthesis components. On the other hand, the positive impact of R light on SSC was possibly due to the expression of key genes being induced, such as *SUS1*. In summary, this research is an integrative and innovative study, where the exploration of the effect of different LED lights on rocket growing under protected cultivation, in a closed chamber cultivation system, was performed at multiple levels.

## 1. Introduction

*Diplotaxis tenuifolia* (rocket) is a native plant of the Mediterranean and belongs to the Brassicaceae family. Subspecies and names of rocket are various and mainly belong to two genera: Eruca and Diplotaxis. Rocket has gained a growing consumer interest in vegetable market. Rocket species are important components of human diets due to their high percentage of health-advantageous substances, such as glucosinolates and phenolics [1], that have many beneficial properties, among them anticarcinogenic effects. *D. tenuifolia* contains several other valuable compounds, including antioxidants (vitamin C) [2]. Different sub-species of rocket are commercially grown in various areas such as USA, Europe, India and Australia [3]. More specifically, rocket consumption as a vegetable is popular in countries such as France and Italy, where it is additionally considered a medicinal plant [4]. 

Light is a key factor in plant development, affecting plant morphology and physiology through its quality, intensity, and photoperiod [5]. Light quality, in particular, is known to affect the activity of the photosynthetic mechanism [6] and root architecture [7] among other parameters. Plants mainly absorb red (600–700 nm) and blue (400–500 nm) wavelengths through pigments such as chlorophylls a and b (mainly), and carotenoids (secondarily). Moreover, protein photoreceptors have been identified, which are activated by light and initiate a signaling cascade leading to plant responses. Phytochromes absorb red and far-red wavelengths; cryptochromes, phototropins and members of the Zeitlupe family absorb blue and UV light; while UVR8 absorbs UV wavelengths [8,9,10]. Nowadays, light quality effects on plants can efficiently be studied with the use of light-emitting diodes (LEDs), a light source with considerable benefits for controlled-environment agriculture [11]. Changes in morphology and physiology of plants due to diverse light conditions cause changes in the gene expression. Transcriptional changes are of paramount importance for plant regulation. A valid technique that allows the detection of genes and low-abundance transcripts is RNA sequencing (RNA-seq). RNA-seq has been used to study the effect of blue light on grape development [12]. Blue light caused the upregulation of microtubule-related genes, serine carboxypeptidase, and genes related to chlorophyll synthesis, while it caused the downregulation of auxin-repressed protein and resistance-related genes. In contrast, red and green light stimulated auxin genes, causing a reduction of auxin concentration, and thus promoting stem and root growth. A study conducted in *Saccharina japonica* seaweed species reported that blue light had an effect on genes related to processes of the circadian rhythm, flavonoid biosynthesis, photo-reactivation, and photo-morphogenesis [13]. Recently, Liu et al. [14] evaluated the effect of supplemental light (green light, G; white light, W; yellow light, Y) added to red–blue light (RB) and sole W light on the growth and photosynthesis of rapeseed *Brassica napus* seedlings. They found that supplemental RB light improved the growth and photosynthesis of seedlings grown inside an incubator and in plastic pots. In sweet basil (*Ocinum basilicum*) and strawberry (*Fragaria* × *Ananassa*), it was found that LED lighting improves yield, energy use efficiency (EUE), antioxidants, and phenolics, while it reduces nitrate concentration; the optimum LED R/B ratio was 0.7, with B light being predominant [15]. Later, it was found that an R/B ratio of 3 (red 70%/blue 23%) was optimal for the sustainable indoor growing of sweet basil, scoring the highest yield, quality, resource (water and energy) use efficiency and antioxidant activity. In the same study it was observed that LED lights with higher blue percentages showed lower yields and chlorophyll content, as well as reductions in resource use efficiency [16]. The same R/B ratio of 3 was proved to promote the sustainable production of vertical lettuce farming, as it increased yield, leaf chlorophyll, and flavonoids [17]. In tomato, increased B light enhances photosynthesis, while R light impairs photosynthetic capacity [18]. In hydroponic kale, on the other hand, it was found that a higher percentage of B light does not have a beneficial effect on the development or nutritional parameters in comparison to W light [19]. Although the effect of different LED lighting on growth, yield and production of secondary metabolites is studied in the literature regarding rocket [20,21], there is still a lack of knowledge on the transcriptome-wide changes that influence gene expression, and hence the manifestation of plant characteristics. In fact, the only wide transcriptomic analysis performed in rocket *D. tenuifolia* concerns transcriptomic changes taking place under pre-harvest and post-harvest stresses that impact the quality and shelf-life [22]. 

Herein, we hypothesized that blue, red, red/blue or white (B, R, R/B or W) LEDs might affect rocket’s adaptive responses in terms of growth traits, photosynthetic and biochemical characteristics. Based on the results, we further explored whether these responses were accompanied by changes at the transcriptional level, using RNA-seq technology. The exploration of the underlying response mechanisms through the identification of differentially expressed genes (DEGs), gene ontology (GO), and Kyoto Encyclopedia of Genes and Genomes (KEGG) enrichment analyses of DEGs will guide the amelioration of lighting technologies and the fine-tuning of indoor cultivation towards sustainable plant production. 

## 2. Materials and Methods

### 2.1. Plant Material and Experimental Design

The experiment was conducted in a controlled environment room (7.14 m length, 2.46 m width, 2.2 m height) at the Institute of Plant Breeding and Genetic Resources of the Hellenic Agricultural Organization, Dimitra, Thessaloniki, Greece (40°32′17.4″ N, 22°59′58.2″ E). The temperature was kept at 21 ± 2 °C, using an air condition unit, relative humidity was fluctuating around 60 ± 10%, using a humidifier, while CO_2_ was monitored, but not controlled. Homogenous air distribution was achieved using air fans in a constant operation. 

Seeds were sown in 275-cell high-density polyethylene trays (30 × 60.5 cm, 1378 plants m^−2^), and kept in high relative humidity (80%), at 22 °C. Upon first true leaf formation (4 days after sowing), plants were placed in the culture tanks, filled with 24 L of a standard Hoagland nutrient solution 100% strength.

### 2.2. Light Treatments 

Four different light treatments were applied. LED lamps were used in three different photon flux ratios of red and blue as follows: 

R:B = 0.3 designated as blue (B)

R:B = 1 designated as red/blue (R/B)

**Figure 1:**
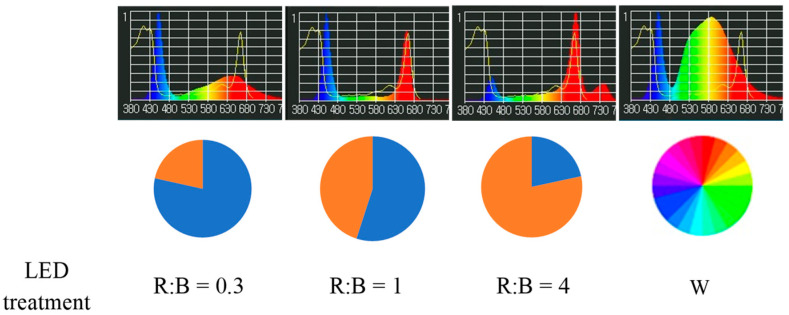
Light spectra used.

R:B = 4 designated as red (R), 

With white light as the control and designated as white (W). Light was provided in a photoperiod of 14/10 h of light/dark (Figure 1). Each lamp was 120 cm in length and was placed horizontally above the plant canopy with a distance, so that the photosynthetic photon flux density (PPFD) could reach 200 ± 10 μmol m^−2^ s^−1^ on the leaf surface. 

Properties, such as photosynthetic photon flux (PPF), Par efficacy, rated power, photosynthetic photon flux density (PPFD), and peak wavelength (λp) of the LED lamps used in the experiment, are presented in Table 1. All LED measurements concerning spectral properties were taken in each shelf separately, using LI-180 Spectrometer (LI-COR Inc., Lincoln, NE, USA). 

### 2.3. Resource-Use Efficiency

All plants were harvested 25 days after first true leaf formation, at the baby leaf stage (about 8 cm leaf length), and the fresh weight was recorded. Recorded data were used for calculating parameters related to resource-use efficiency.

Water-use efficiency was defined as a plant’s fresh weight per total amount of water supplied to the culture tanks and absorbed by plants (g Fw L^−1^ H_2_O) [15].

Energy-use efficiency, respectively, was calculated as the ratio between total yield and total electricity consumption [15]. In order to determine the electrical energy consumption, own measurements, based on energy data logger, as well as the manufacturer’s information of the rated power, were used.

Land-use efficiency was calculated based on the potential annual yield per square meter (kg m^−2^ a^−1^) [17]. Therefore, in our case, yield was calculated considering a cultivation period of 25 days, leading to approximately 14 cycles per year in two scenarios, featuring a single layer or a vertical layout with four layers. 

### 2.4. Morpho-Physiological Measurements

The LICor Plant Canopy Analyzer LAI-2200C used for the non-destructive leaf area index (LAI) assessment in situ, measuring diffuse radiation underneath the canopy and measurements above the canopy, served as a reference [23]. 

At the end of the experimental period, rocket was harvested for the determination of developmental and physiological characteristics. Specifically, leaf mass per area (i.e., yield) was determined by weighing all plants in each tray. Leaf length and leaf area were measured with a digital caliber and a leaf area meter (LI-3000C, LI-COR, HΠA), respectively, while leaf number was also measured, all in 10 plants per light treatment. Shoot dry weight was determined in 10 plants per light treatment after oven-drying (72 °C) them for three days. Relative chlorophyll content was determined with a CCM-200 plus (Opti-Sciences, Hudson, NH, USA), while colorimetry was conducted with a CR-400 Chroma Meter (Konica Minolta Inc., Tokyo, Japan), and the parameters obtained were lightness (L*), chroma (C*), hue angle (h°), and a* and b* coordinates. Briefly, L* refers to the lightness of the leaf, hue parameter is the visible color, C* represents color saturation, a* corresponds to the red/green coordinate, and b* corresponds to the yellow/blue coordinate.

Moreover, roots from 5 seedlings per light treatment were thoroughly rinsed, scanned, and analyzed using the WinRHIZO Pro software (Regent Instruments Inc., Québec, QC, Canada). Specifically, the analyzed parameters of the root architecture were root length, surface area, average diameter, and the number of tips.

### 2.5. Biochemical Analysis

For the determination of soluble sugar content (SSC), total phenolic content (TPC), antioxidant capacity, and nitrate content, rocket leaves were macerated in a blender and mashed in a mortar. SSC was measured in a few drops of the pulp with a refractometer (PAL-α, Atago, Tokyo, Japan). For the determination of TPC, 80% methanolic plant extract (0.5 mL), Folin–Ciocalteau reagent (2.5 mL), and 7.5% sodium carbonate solution (2 mL) were incubated for 5 min at 50 °C, while the absorbance of the colored product was determined at 760 nm, according to [24]. A ferric-reducing antioxidant power (FRAP) assay was used for antioxidant activity determination. A mix of TPTZ, FeCl_3_, and CH_3_COONa buffer solution (3 mL) was added to 80% methanolic plant extract (0.1 mL), and incubated for 4 min at 37 °C, while the absorbance of the colored product was determined at 593 nm, according to [25]. For the determination of nitrate content, aqueous plant extract (0.2 mL) was mixed with H_2_SO_4_ or 5% salicylic acid in H_2_SO_4_ (0.8 mL), and 2N NaOH (19 mL) was added, while the absorbance of the colored product was determined at 410 nm, according to [26].

### 2.6. Chlorophyll Fluorescence Measurements 

Chlorophyll fluorescence was measured in 20 min dark-adapted *D. tenuifolia* leaves, using an Imaging-PAM fluorometer M-series MINI-version (Walz, Effeltrich, Germany), as described by [27]. Six leaves from six different plants were measured from each light treatment, and two actinic light intensities were selected: a low light (LL) of 200 μmol m^−2^ s^−1^, in order to match that of the growth conditions, and a high light (HL) of 1000 μmol m^−2^ s^−1^. The chlorophyll fluorescence parameters measured were the maximum efficiency of photosystem II (PSII) photochemistry (Fv/Fm), efficiency of the water-splitting complex on the donor side of PSII (Fv/Fo), effective quantum yield of PSII photochemistry (ΦPSII), quantum yield of regulated non-photochemical energy loss in PSII (ΦNPQ), quantum yield of non-regulated energy loss in PSII (ΦNO), non-photochemical quenching that reflects heat dissipation of excitation energy (NPQ), and the excess excitation energy (EXC) [28].

For the statistical analysis, chlorophyll fluorescence parameters represented averaged values from two independent experiments with six leaf samples (each leaf sample from a different plant) per treatment, per experiment. Results are expressed as the mean ± standard error (SE). Statistically significant differences between the treatments were analyzed by the Student’s *t*-test at a level of *p* < 0.05 (StatView computer package, Abacus Concepts, Inc Berkley, CA, USA). 

### 2.7. RNA Isolation and Transcriptome Sequencing 

For the isolation of total RNA, three independent biological replicates of each rocket salad tissue were frozen in liquid nitrogen and stored at −80 °C. RNA was isolated using the TRIzol^®^ reagent (Thermo Fisher Scientific, Waltham, MA, USA), according to manufacturer’s instructions. 

The RNA concentration and integrity were assessed using the RNA Nano 6000 Assay Kit of the Agilent Bioanalyzer 2100 system (Agilent, Santa Clara, CA, USA) to ensure that the RNA integrity number (RIN) values were above 7.0. Equal amounts of RNA samples from the three independent biological replicates (of each tissue) were pooled prior to library preparation. Oligo (dT) beads were used to isolate poly (A) + mRNA, which was fragmented to 250 bp. Each cDNA library was sequenced in a single lane of the BGISEQ-500 system, with a paired-end sequencing length of 100 bp, according to the manufacturer’s instructions, at the Beijing Genomics Institute (BGI-Shenzhen, Denmark).

### 2.8. De Novo Assembly, Annotation and Analysis 

For the quality of assembly, clean reads were obtained by removing reads containing adapter, reads containing ploy-N and low-quality reads from the raw data. The Trinity method was employed to de novo assemble the clean reads [29]. Seven public databases or programs were used to annotate the genes, including the NCBI non-redundant protein (Nr), the NCBI nucleotide sequences (Nt), the protein family (Pfam), the euKaryotic ortholog groups (KOG), the Swiss-Prot database, the Kyoto Encyclopedia of Genes and Genomes (KEGG), and gene ontology (GO). We used Blastn (v2.2.23) or Diamond (v0.8.31) to align Unigenes to NT, NR, KOG, KEGG, and SwissProt databases to do the annotation. Blast2GO (v2.5.0), with NR, annotation was used to perform GO annotation, and InterProScan5 (v5.11-51.0) to do the InterPro annotation.

The fragments per kilobase of transcript per million mapped transcript (FPKM) values were used to analyze gene expression. The differential expression analysis of any two sets of treatments was measured using the R package DEGseq2 [30]. An adjusted *p*-value of 0.05 was set as the threshold to determine significant differences in DEGs. The enrichment analysis was performed with GO, using the R package GOseq [31]. The KEGG pathway was annotated using the KEGG database. The corrected *p*-value < 0.05 and |log2 (FoldChange)| ≥ 1 were set as the threshold to determine the significant differences under the GO and KEGG enrichment analyses. Heatmaps were generated using TB tools [32]. 

### 2.9. Validation of RNA-seq Data by qRT-PCR 

Eight genes with significant differences in RNA-seq-based expression values between treatments were analyzed by qRT-PCR. First-strand complementary DNA (cDNA) synthesis was performed using the same RNA samples (500 ng) used for RNA sequencing, and isolated from three independent biological replicates, consisted of three plants each, but without pooling, using the LunaScript^®^ RT SuperMix Kit for cDNA synthesis (NEB), according to the supplier’s protocol. Gene expression profiles were analyzed using the Luna^®^ Universal One-Step RT-qPCR Kit (NEB) in the QuantStudio 5 real-time PCR system (Applied Biosystems, Waltham, MA, USA), following manufacturer’s instructions.

### 2.10. Statistical Analysis

Statistical analysis of the morpho-physiological and biochemical determinations was performed with the IBM SPSS software (SPSS 23.0, IBM Co., Armonk, NY, USA). Data were analyzed with one-way analysis of variance (ANOVA), at a significance level a = 0.05. Mean comparisons were conducted using a Tukey’s test at a < 0.05.

## 3. Results

### 3.1. Physiological and Biochemical Data Analysis 

The dimensionless leaf area index (LAI) of horticultural crops is an important characteristic, related directly to canopy photosynthesis. Furthermore, LAI is an indication of transpiration and how much light is passing through the canopy. In the current study, the highest value of LAI was observed under W light (3.11), followed by the R/B (2.67), R (2.36), and finally B light (2.15), however ANOVA showed no statistically significant differences. 

Leaf area and leaf number were not significantly affected by the different light treatments. However, leaf length was greater under R (79.6 mm) and W (83.1), compared to R/B wavelength (55.1). Relative chlorophyll content was also unchanged by the different wavelengths tested. Shoot fresh and dry weight were also not significantly affected by the different light treatments. Among the colorimetric parameters, chroma was significantly different between W (24.03) and R/B (17.11) wavelengths.

Regarding the root architecture, total root length, root surface area and tips were not affected by the different light treatments. However, the average root diameter was greater in plants treated with R compared to B light (data not shown).

**Figure 2:**
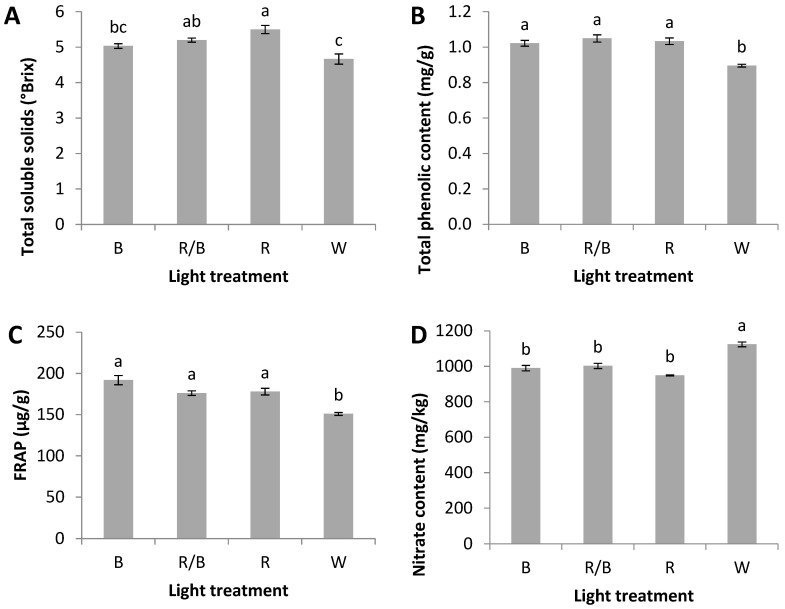
(**A**) Total soluble solids, (**B**) total phenolic content, (**C**) antioxidant activity displayed by ferric reducing antioxidant power (FRAP), and (**D**) nitrate content of baby rocket leaves cultivated in a PFAL system under four light treatments, described in Figure 1. Bars (±SE) followed by the same letters denote no statistically significant differences.

Total soluble solids were significantly enhanced under R (5.5° Brix) compared to W (4.7) and B (5.0) (Figure 2). Moreover, the total phenolic content and the antioxidant activity displayed by FRAP were also significantly greater under R (1.0 mg/g FW and 177.9 μg/g FW) compared to W (0.89 and 150.9). On the contrary, nitrate content was higher in leaves treated with W (1124.2 μg/g FW) compared to the rest of the light treatments (Figure 2).

### 3.2. Resource-Use Efficiency 

Water-use efficiency was slightly increased from R (4.59 g Fw L^−1^ H_2_O) to R/B (5.28 g Fw L^−1^ H_2_O), while the increase was greater in comparison to B (7.22 g Fw L^−1^ H_2_O) and W lights (7.78 g Fw L^−1^ H_2_O) thanks to higher fresh weight production and lower water consumption, comparing to the first two.

Energy-use efficiency follows the same path. W LED light showed the best results (11.79 g FW kWh^−1^) due to a higher yield and lower energy consumption, while R (5.54 g FW kWh^−1^) was still not as efficient as the rest regarding energy consumption.

Land-use efficiency, in both scenarios of one and four layers, agreed with the rest of the efficiencies, since it was directly connected to fresh weight. Consequently, W and B LED lights showed the best results, R/B followed, with slightly less difference, while R LED lights came last. All results are shown in Table 2. Despite the differences mentioned above, ANOVA statistical analysis showed that the differences recorded among light treatments were not statistically significant for a significance level a = 0.05 (Figure S1).

### 3.3. RNA Sequencing, De Novo Assembly and Annotation of the Reference Transcriptome 

Messenger RNA (mRNA) sequencing of the leaf samples, after 15 days of lighting, was performed, with the aim to enlighten the molecular mechanisms behind supplemental light responses. The 12 cDNA libraries constructed, produced a total of 790.5 million high-quality clean reads, and 864,4 million raw reads. Each library comprised of 66.98–72.46 million raw reads. Functional annotation analysis, through seven public databases, revealed that 102,035 (NR: 78.95%), 97,850 (NT: 75.71%), 75,784 (Swissprot: 58.64%), 82,635 (KOG: 63.94%), 76,460 (KEGG: 59.16%), 57,899 (GO: 44.80%), and 77,890 (InterPro: 60.26%) unigenes were annotated.

### 3.4. Differential Gene Expression and Gene Ontology (GO) Enrichment of DEGs under Different Light Treatments 

**Figure 3:**
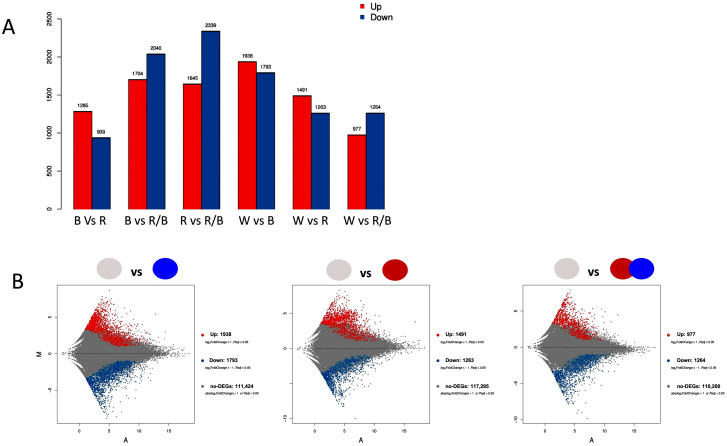
(**A**) Pairwise comparisons of DEGs in different light conditions. Red coloring denotes the upregulation, while blue coloring the downregulation of genes. *y*-axis represents the number of up- or downregulated DEGs, (**B**) MA plots of DEGs; the *x*-axis signifies value A (log2 transformed mean expression level), while the *y*-axis signifies value M (log2 transformed fold change). Again, red colored dots are the upregulated DEGs, while blue dots are the downregulated DEGs. Black dots are the non-DEGs.

Pairwise comparisons revealed that the highest number of DEGs was recorded between R and R/B light (3984) the majority of which were downregulated in R light. A high number of DEGs were also recorded in B vs. R/B (3744). The lowest number of DEGs was noted between B and R light (2224), most of them being upregulated by B light (Figure 3A). When compared with W light, B light had the highest number of DEGs (3731) in comparison to R (2754) and R/B (2241) (Figure 3B). The highest number of upregulated DEGs was documented in W vs. B light, while the highest number of downregulated DEGs was documented in R vs. R/B light. 

In order to decrypt the expression of DEGs, and consequently gain a better understanding of the molecular, biological and cellular mechanisms that govern gene expression, a DEG enrichment analysis was conducted. The enrichment analysis was applied through GO analysis (Spreadsheet S1). Plurality of DEGs was found to be involved in six categories: “cellular process”, “metabolic process”, “ion binding”, “structural molecule activity”, and “catalytic activity”. 

Τhe “biological process” category comprised 3355 (W vs. B), 2522 (W vs. R), and 1644 (W vs. R/B) DEGs. In W vs. B, the DEGs were mainly involved in “cellular process”, “metabolic process”, and “response to stimulus” categories; half of the DEGs were downregulated, and the other half were upregulated. In W vs. R, the DEGs were involved in “biological regulation”, “cellular component organization or biogenesis’, “cellular process”, “developmental process”, “metabolic process”, “multicellular organismal process”, “regulation of biological process”, and “response to stimulus”; half of the DEGs were downregulated, and the other half were upregulated. In W vs. R/B, the DEGs were involved in “cell proliferation”, “detoxification”, “rhythmic process”, and “carbon utilization”, with the exception of “rhythmic process”, which was primarily upregulated by R/B; these terms were all mainly upregulated by W light.

**Figure 4:**
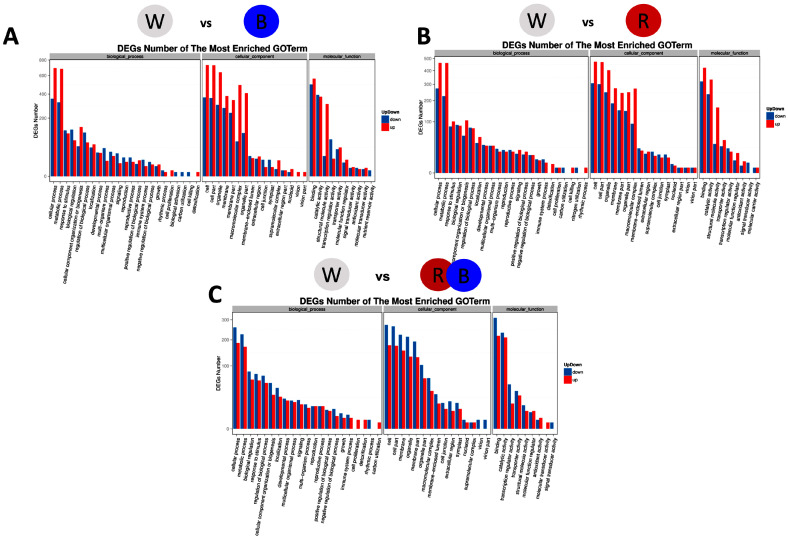
GO classification of upregulated and downregulated genes in B (**A**), R (**B**) and R/B (**C**) lights, in comparison to W light.

DEG enrichment analysis found 2403 (W vs. B), 1648 (W vs. R), and 1146 (W vs. R/B) DEGs involved in the “molecular function” category (Figure 4). In W vs. B, W vs. R and W vs. R/B, the DEGs were involved in “binding” and “catalytic activity” categories, and half of the DEGs were downregulated, and the other half were upregulated.

### 3.5. Candidate Genes Involved in the Phenylpropanoid Biosynthesis

Herein, the transcript expression levels of most of the main genes involved in phenylpropanoid pathway, as suggested by [33], were downregulated in B, R and R/B lights when compared to W light. The highest number of the most expressed genes was recorded under R light (16 genes), followed by R/B light (12 genes). On the contrary, the highest number of the least expressed genes was recorded under B light (18 genes). R light upregulated the expression of an *FLS* gene (CL14819.Contig2). Significant differences were also noted for a *CCoAMT* gene (Cl2138.Contig1), which was significantly expressed more in W light and in R/B light, in comparison to B. Large differences were also recorded in two *C4H* alleles (CL8338.Contig5 and CL8338.Contig10), expressed more in R light; in the *FLS1* gene (Cl2235.Contig1), expressed more in B light; and in the *ALDH* allele (CL15019.Contig3), which was significantly more expressed in R light. Genes that were less expressed in W light (the light where the lowest phenolic content was recorded) were CL8338.Contig5 (*C4H*) and CL5158.Contig3 (*CHS3* allele). 

*PAL*, *CHS*, *CHI* and *F3H* are considered key genes in the phenylopropanoids pathway. However, among these, only two genes were differentially expressed significantly: *CHS* was induced by R/B light, and *F3H* was induced by R/B and B lights. In general R light induced the expression of some key genes, although there was not a universal trend on the impact of lighting on the expression of genes participating in the phenylpropanoid pathway.

### 3.6. Chlorophyll Fluorescence Analysis under Different Light Treatments

**Figure 5:**
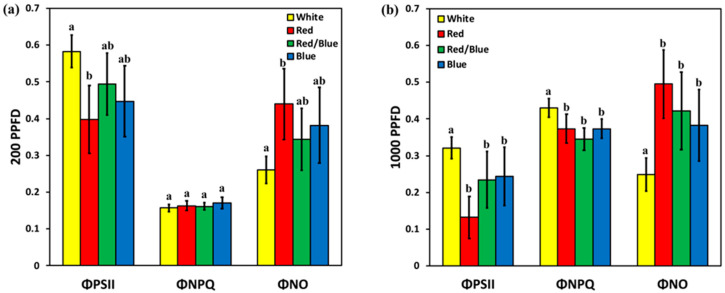
Changes in the effective quantum yield of PSII photochemistry (ΦPSII), quantum yield of regulated non-photochemical energy loss in PSII (Φ_*NPQ*_), and the quantum yield of non-regulated energy loss (Φ_*NO*_) in rocket leaves, under different light treatments: (**a**) in the low light (LL) 200 μmol m^−2^ s^−1^, and (**b**) the high light (HL) 1000 μmol m^−2^ s^−1^. Error bars are standard errors. Columns with different letters are statistically different (*p* < 0.05).

By measuring the effective quantum yield of PSII photochemistry (Φ*_PSII_*), the quantum yield of regulated non-photochemical energy loss (Φ*_NPQ_*) and the quantum yield of non-regulated energy loss (Φ*_NO_*), we estimated the balance between light capture and photochemical energy use in rocket leaves, under four different light treatments. These three quantum yields added up to unity [34]. Under W light, rocket leaves retained a higher Φ*_PSII_* and a lower Φ*_NO_* compared to R light in low actinic light (LL, 200 μmol m^−2^ s^−1^), although they were not significantly different compared to R/B and B light (Figure 5a). In LL, Φ*_NPQ_* did not change under any light treatment (Figure 5a). In high actinic light (HL, 1000 μmol m^−2^ s^−1^), Φ*_PSII_* and Φ*_NPQ_* increased significantly under W light compared to all the other light treatments, and that resulted in a significantly lower Φ*_NO_* (Figure 5b). There was not any significant difference between R, B and R/B light in the three quantum yields, nor under LL or HL (Figure 5a,b). 

**Figure 6:**
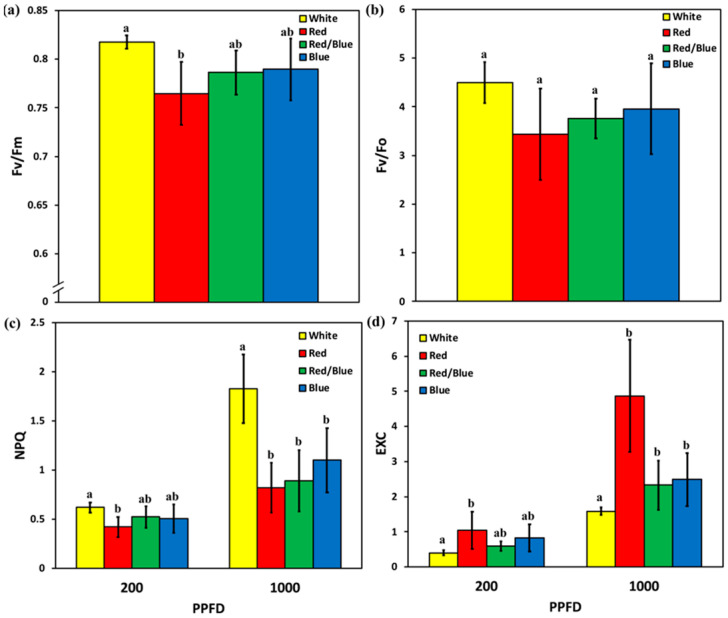
Changes in (**a**) the maximum efficiency of PSII photochemistry (Fv/Fm), (**b**) the efficiency of the water-splitting complex on the donor side of PSII (Fv/Fo) in rocket leaves, under different light treatments, and (**c**) non-photochemical quenching (NPQ). (**d**) The excess excitation energy (EXC) in rocket leaves, in the low light (LL) 200 μmol m^−2^ s^−1^, and the high light (HL) 1000 μmol m^−2^ s^−1^, under different light treatments. Error bars are standard errors. Columns with different letters are statistically different (*p* < 0.05).

Moreover, we calculated the maximum efficiency of PSII photochemistry (Fv/Fm) and the efficiency of the water-splitting complex on the donor side of PSII (Fv/Fo). Fv/Fm was significantly increased under W light compared to R light that had the lowest value (Figure 6a), and no significant difference was observed for Fv/Fo under any treatment (Figure 6b). The non-photochemical quenching (NPQ) that reflects heat dissipation of excitation energy showed a significant difference between W and R light in LL, with the latter having the lowest value among all treatments, while in HL, W was significantly increased compared to R, R/B, and B light, indicating high photoprotection (Figure 6c). Finally, excess excitation energy (EXC) was significantly different between W and R light in LL (Figure 6d), while in HL, EXC was significantly decreased under W light compared to all the other treatments (Figure 6d). 

### 3.7. Transcriptomic Analysis of Genes Encoding Photosynthesis Components 

#### 3.7.1. Photosystem II

**Figure 7:**
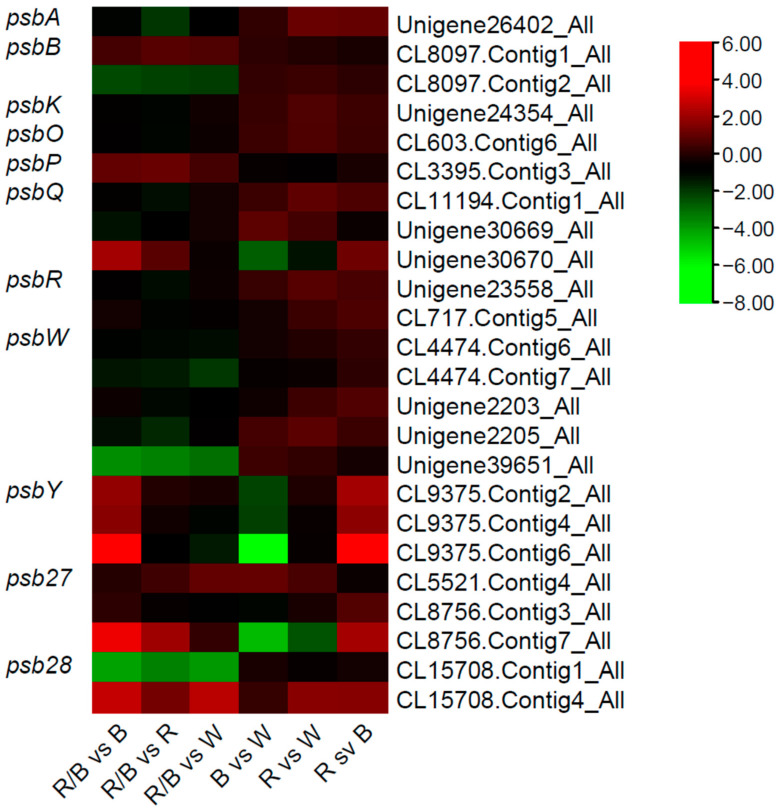
Heatmap illustrating differentially expressed genes encoding protein components of the photosystem II.

Analysis of genes that encode proteins that participate in the photosystem II protein complex, has shown that most of the DEGs were recorded under R/B LED light (total of 33 genes). However, the majority of these DEGs were inhibited (24 genes). R light had the most profound positive effect on the transcription of genes, followed by W light, since 17 and 12 genes, respectively, were found to be significantly upregulated. An overview of the differentially expressed gene-encoding components of PSII is shown in Figure 7.

Looking deeper into the heatmap and the genes that encode for proteins of PSII, there are some interesting results. First, there are genes significantly affected by R/B light. A *psbA* gene (photosystem II P680 reaction center D1 protein) homolog, Unigene26402, was significantly downregulated under R/B light. The same gene was upregulated in R light. PSII oxygen-evolving enhancer protein 3 (psbQ) gene homolog, CL11194.Contig1, was found to be significantly downregulated by R/B light and upregulated under R light. Alleles of a putative photosystem II reaction center W gene (protein *B. napus* psbW), CL4474.Contig6 and CL4474.Contig7, were significantly downregulated in R/B light (vs. all other lights). These genes were upregulated by W light (vs. all other lights). Another PSII reaction center W homolog, Unigene39651, was also significantly downregulated by R/B light. There was also a negative influence of R/B on CL15708.Contig1, and a positive effect on CL15708.Contig4 (psb28 homologs *B. napus* XP_013707552), which is contradictory.

Secondly, there are genes significantly influenced by W light. CL9375.Contig2, CL9375.Contig4, and CL9375.Contig6 were enhanced by W light. All three alleles were upregulated in W light vs. all other lights, but the largest differences were recorded in W vs. B light. These genes were encoding a putative photosystem II psbY protein. Unigene30670, encoding a psbQ-like protein, was also upregulated in W light (vs. all other lights).

Finally, there were genes affected by B light. The most profound effect was recorded on the expression of CL8756.Contig7. The gene was significantly downregulated by B light while it was upregulated by W light (W vs. B and W vs. R), in R vs. B and R/B vs. B. CL8756.Contig7 is a psb27-H1 putative ortholog gene, encoding for a protein that is highly similar to the B. rapa protein (NCBI: XP_009111150). CL8756.Contig3, that is also coding for another protein highly similar to photosystem II repair protein, PSB27-H1, from *B. rapa* (XP_009119529), was only positively regulated in W vs. B, while CL5521.Contig4, a putative photosystem II D1 precursor processing protein PSB27-H2 (XP_022558380), was not affected in the same way by LED lights. This gene was only slightly upregulated in R/B vs. W and in B vs. W. Lastly, under B light, CL9375.Contig6, a *psbY* gene, was significantly downregulated. 

As for Cytochrome b6/f complex proteins, CL14627.Contig1 and CL14627.Contig2 encoding for petA (apocytochrome f) protein, and were both found to be downregulated by R/B light. The same result was observed for petN and petG homologs that were also downregulated in R/B light. *Ferredoxin C1* gene homolog, CL13647.Contig1, was significantly upregulated in R and R/B lights.

In total, R/B vs. R light was the comparison with the most DEGs (19 genes up- or downregulated), followed by R/B vs. B light (18 genes). The most downregulated genes were observed in the R/B vs. R and R/B vs. B light. This supports the notion that R/B light is negatively influencing the expression of genes participating in the photosystem II, cytochrome b6/f complex and photosynthetic electron transport. R light is promoting the expression of more genes than any other light. 

#### 3.7.2. Photosynthesis: Antenna Proteins

B light induced the expression of more genes encoding for antenna proteins. On the other hand, R/B light downregulates the expression of more genes than any other light. 

CL10563.Conti8 is upregulated under B light. However, the impact on CL10563.Contig4, another *Lhca4* allele, is more prominent: the gene was significantly downregulated in R/B vs. B (log2fold change −10.17) and R/B vs. W (−8.7). This gene is a chlorophyll a–b binding protein 4, coding gene *Lhca4* (*B. rapa*). *Lhcb7* genes are also affected. CL470.Contig 4 is upregulated under W light.

### 3.8. Effects of LED on Quality: Soluble Solid Content 

**Figure 8:**
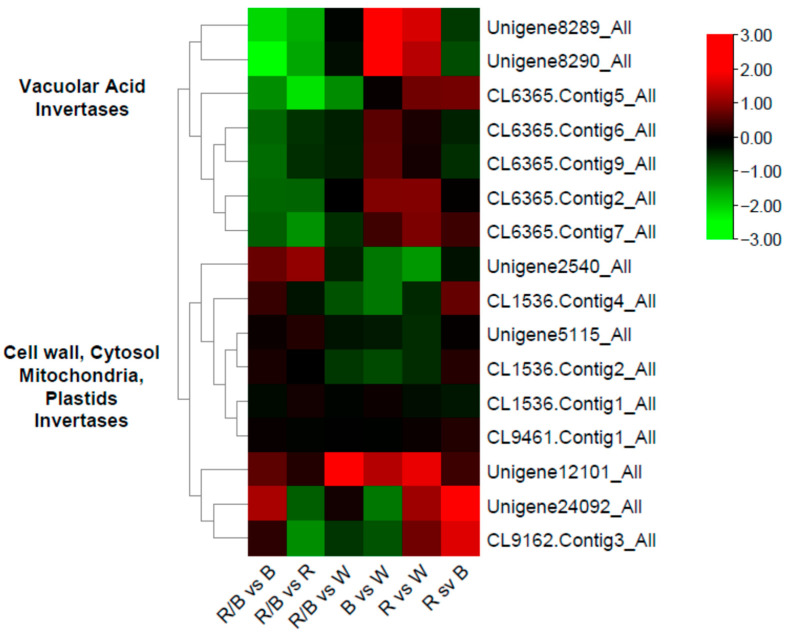
Heatmap illustrating differentially expressed genes encoding invertases, and their euclidean distances. Vacuolar acid invertases seem to be co-expressed (common expression motifs across light comparisons). Cytosol, mitochondria, plastids invertases, which are neutral/alkaline, are co-expressed too. One cell wall invertase which is also an acid invertase is included in the same branch with the alkaline/neutral invertases.

Sucrose synthase genes (*SUS*) are catalyzing the reversible conversion of sucrose to fructose and UDP glucose, hence their role in sugar catabolism is vital. In our transcriptomic data, we have found a strong induction of a gene encoding a SUS1 protein (*B. rapa* XP_013667258) homolog; the gene is upregulated under R light (in comparison to all other lights) (Figure 8). Another paralog is only upregulated significantly when R light is compared to R/B light. Given that rocket plants have a significantly higher soluble solid content (SSC) under R light in comparison to B, R/B and W lights, there is a strong indication that our *SUS1* genes control this induction. The strong correlation between the expression of *SUS1* genes and SSC (r = 0.922 and r = 0.817, respectively) supports this notion. Furthermore, there is a potent influence of R light on the expression of several invertases. Invertase is the enzyme that controls the irreversible hydrolysis of sucrose to glucose and fructose, while it exists in the cell wall and vacuole as acid invertase, and in the cytosol, mitochondria and plastids, as neutral/alkaline invertases. R light induces the expression of a putative cell wall *CWINV1* (acid invertase), which in contrast is downregulated in B light. Two more acid invertases (CL6365.Contig 5 and CL6365.Contig7 encoding for an acid beta-fructofuranosidase 4) located in the vacuole are significantly downregulated by R/B light and upregulated by R light, while another one, Unigene8290, is upregulated by B light. A putative *neutral invertase* (Unigene12101) is downregulated by W light. There is not a clear trend on the expression of invertases, but the upregulation of three vacuolar acid invertases by R light might relate to the enhanced soluble solid content (Figure 8).

### 3.9. Validation of RNA-Seq Results Using qRT-PCR 

Τo confirm the results of RNA-seq data analysis, quantitative real-time polymerase chain reaction (qRT- PCR) was applied, assessing the gene expression of eight randomly selected DEGs. qRT-PCR expression analysis was employed to validate the RNA-seq results of four DEGs, which were selected participating in the pathways of interest. Results showed that in qPCR analysis, the expression levels of the selected genes had similar patterns with the expression identified by RNA-seq data analysis (FPKM), confirming the validity of the RNA-seq results (Figure S2).

## 4. Discussion

Light is essential for plant growth, photosynthesis, and development. However, the impact of light on the development of plants is not simple; apart from controlling growth, light also affects flowering and morphogenesis. Photoreceptors such as phytochromes and cryptochromes, that absorb red/far-red light and blue/ultraviolet light, respectively, are the main modulators of various and crucial aspects of biological activity [35]. 

LED lighting is becoming a standard practice in modern indoor agricultural cultivation, because using such lighting, the intensity and composition of the light spectrum can be efficiently controlled, while less energy is consumed. In general LEDs enhance synthesis of bioactive compounds, such as the plant primary and secondary metabolites, along with antioxidant properties, and ameliorate nutrient content and post-harvest qualities and life, potentially maximizing the crop yield [36]. 

Red light has been associated with the maximum absorption of chlorophyll pigments, while blue light induces the opening of the stomata, leading to a greater CO_2_ fixation and biomass accumulation. Blue or red LEDs have been associated with enhanced quality and yield in cucumber, pepper, lettuce, spinach, and strawberry [37,38]. In lettuce, fluorescent light, coupled with red and blue LEDs, improved morphology and the biomass of the “Green Oak Leaf” variety [39]. However, it is still largely unknown how the LED light affects other important vegetable crops, such as rocket. 

For the purpose of studying the response and adaptation of rocket plants to different LED lighting conditions, we evaluated the effect of B, R and R/B light on the morphology, physiology, biochemistry and leaf transcriptome of rocket plants, and compared it to standard white LED lighting. We discussed whether treating plants with these different LED conditions has a strong impact on resource-use efficiency, water-use efficiency, or land-use efficiency, and which LED treatment was the most efficient. 

In terms of yield, R light produced the least in comparison to other LED lights, although differences in fresh and dry weight were not significant. This comes in contrast to what was previously found in lettuce, where the more R added to the light treatments, the more the fresh lettuce weight was, with RB = 3, producing maximum yield [17]. However, it was recently observed that two varieties of lettuce yielded more (fresh weight) under white (control) light than under the combination of R/B light, or R/B light supplemented with G or far-red lights [40]. In rocket *Eruca sativa* Mill., it was observed that a combination of R, G and B lights (50%: 20%: 30%) had the highest dry weight [20]. As R light is known to promote photosynthesis, it should be expected to cause a higher yield in rocket, as it was recently found by [21]. However, in our experiments, differences in yield were not statistically significant, therefore different LED lights did not affect yield considerably. Still, the impact of different LED lighting on yield is more complex. In lettuce, it was found that different light wavebands during different growth stages are needed to achieve an increase in plant biomass [41]. In lettuce, kale, pepper, spinach, and basil different percentages of B and R LED lights are needed for the increase of growth characteristics, pigment content and antioxidant activity, while the response is species-dependent [42]. Other studies have highlighted the species-dependency of LED effects [43]. Viršilė et al. (2020) have also found that dynamic lighting in lettuce is cultivar-dependent (red vs. green leaf lettuce), and that the imitation of natural light fluctuations did not have an impact on photosynthesis, nor antioxidative response [44]. Ιn a study with two basil cultivars, shoot biomass was variably affected by four broad-spectrum LEDs, depending on slight differences in the radiation spectrum of each light fixture, with the “more white” spectra generally exhibiting greater values compared to the “more red” spectra [45].

In the context of physiology, chlorophyll fluorescence serves as a reflection of the photochemical activities of photosynthetic systems in plant physiological experiments [46]. In the present study, ΦPSII and ΦNPQ were significantly increased in plants grown under W light and HL, compared to all the other light treatments (Figure 6b), and they therefore possessed the lowest ΦΝO, implying a better photoprotection and indicating a lower 1O2 production [47,48]. The inability of PSII to utilize the absorbed light energy for photochemistry (ΦPSII), or to safely dissipate it as heat (ΦNPQ), can lead to the formation of a triplet chlorophyll state (3Chl*) that can react with O2 to produce the very reactive 1O2 [49]. The probability of formation of 3Chl* can be calculated with ΦΝO [50]. An increased ΦΝO reflects the inability of a plant to protect itself against damage by excess illumination, which would eventually lead to photodamage [51], suggesting that the plant has problems coping with the incident HL [38]. Our results come in accordance with [52], who pointed out that *Cyclocarya paliurus* plants performed better, regarding PSII activity, under W light treatment compared to R, B and G light. Moreover, in our study, R light enhances the expression of *psbA*, especially over W light, possibly due to a low quantum yield of PSII photochemistry. Thus, rocket plants try to maintain a higher photosynthetic capacity, as [53] also revealed in their results concerning lettuce grown under R light. Improved photosynthetic efficiency is achieved via a better allocation of absorbed light energy that also reduces photooxidative stress [54].

It seems that the light quality had no effect on the efficiency of the oxygen-evolving complex. In contrast, plants under R light presented lower values of maximum efficiency of PSII photochemistry (Fv/Fm) compared to W light, suggesting a higher degree of photoinhibition [48,55]. 

Photosystem II proteins are numerous, including reaction center core proteins D1 and D2 (also known as PsbA and PsbD), core antenna proteins cp43 and cp47 (PsbC and PsbB), cytochrome b559 subunits alpha and beta (PsbE and PsbF), and low molecular mass (LMM) proteins PsbH, PsbI, PsbJ, PsbK, PsbL, PsbM, PsbR, PsbTc (chloroplast-encoded PSII protein T), PsbTn (nuclear-encoded PSII protein T), PsbW, PsbX, PsbY, and PsbZ. PSII also contains oxygen-evolving complex (OEC) proteins, PsbO, PsbP and PsbQ subunits [56]. Psb27 protein plays a significant role in the assembly, preservation, and stability of PSII [57,58], while it was recently found that it has a significant role in Arabidopsis by permitting plants to adapt to a changing light intensity [59]. Psb28’s role is still under investigation. In the current experiment, the most differentially expressed genes encoding for photosystem II components were observed under R/B light. The effect was mostly inhibitory, while R light had the opposite effect by upregulating most of the genes of photosystem II, in comparison to other light treatments. Contrary to our results, in *Camelia sinensis*, a yellow leaf tea cultivar sensitive to light, it was found that most of the genes encoding psaB, psbA, PSI and PSII components were impaired by R light compared to W light, although they were upregulated under B light. However, under R/B light, five crucial photosynthesis-related transcripts, such as genes encoding for PSI, PSII, *Lhca*, and *Lhcb*, were downregulated in comparison to W light [60], agreeing partly with our results where R/B light inhibited most genes in the PSII pathway. 

Furthermore, a *PsbA* gene homologue was significantly upregulated under R light and downregulated by R/B light. Monochromatic green, blue of red light were recorded to have no significant impact on the psbA mRNA abundance in Arabidopsis, while supplemental UV-A light significantly enhanced D1 synthesis and the recruitment of psbA in ribosomes [61]. We showed that R light enhances the expression of *psbA*, especially over W light, but that the equal mix of R and B light (R/B) did not induce the transcription of *psbA* in rocket leaves. Enhancement of *psbA* expression could be important for promoting rocket’s photosynthesis, as it was recently shown that the overexpression of a maize *psbA* gene in tobacco improves photosynthesis under drought stress by upregulating the expression of stress-defense genes and enhancing the activities of antioxidant enzymes [62]. Rocket plants growing under R/B light could have a disadvantage due to the negative influence of R/B light on *psbA*. However, R/B light positively regulates the expression of Psb27 homologs, in rocket leaves. In the Arabidopsis genome, there are two *Psb27* homologues: *Psb27-H1* and *Psb27-H2*. *Psb27* homologs seem to play different roles in PSII biogenesis and repair in Arabidopsis. *Psb27-H2* Arabidopsis mutant analysis has proven that the protein is important for PSII biogenesis and stabilization in such a way that gene mutation results in inhibition of PSII content and growth. On the contrary, the role of *Psb27-H1* is not vital [63]. *Psb27-H1* was found to be essential after photoinhibition, working on the repair of damaged PSII [64]. The fact that in our data, *Psb27-H1* and *Psb27-H2* are negatively regulated by B light, might suggest that this lighting might lack in PSII content. R/B light significantly enhances the expression of *psbW* homologs. There are numerous reports on the functional role of *psbW* in the formation and stabilization of PSII–LHCII super-complexes [65]. In general, there seems to be no universal trend on how lighting collectively affects PSII components, but the specific impact of R and R/B on some components might be of special interest. In the recent literature, it is found that the effect of LED lighting on plants could often be even cultivar-specific [66]. 

Antioxidant capacity increases it the presence of oxidative stress. Under W light, plants possessed the lowest excess excitation energy (EXC), revealing low ROS production, and therefore no need for antioxidant capacity. In addition, we found that the high photoprotective capacity of plants grown under W light resulted in a low EXC under HL. Photoprotection by non-photochemical quenching (NPQ) is important for optimal growth and development [56], as shown by the highest fresh weight production for plants under W light. NPQ mechanism is often measured to identify the adaptation level of plants under light stress, as they can effectively reduce the damage caused by excessive light energy [67,68]. 

Plants respond to environmental stress factors, such as increased salinity, drought, and excess light, by producing and accumulating antioxidant compounds as a means of adaptation to these conditions. Vegetables, in particular, are essential for human health, since they regulate the oxidative stress by providing a number of antioxidant compounds [69]. Phenolics are especially among the compounds exerting the highest antioxidant activity involved in plant defense and signaling [70], and are regulated by light quality [71]. In this study, the accumulation of both antioxidant compounds, as displayed by FRAP assay, and total phenolic compounds were inhibited by W light compared to the LED wavelengths. In a study with strawberry, fruits showed a greater FRAP under 1.5 and 5.5 red/blue ratios compared to 0.7 [15]. An experiment involving microgreen vegetables also revealed a significantly lower FRAP and total phenolic content under a similar treatment to our W light, in five and six microgreens, respectively [72]. In another study with purslane, plants grown under W light produced a lower amount of phenolics compared to an R/B treatment [73]. Phenylalanine ammonia lyase (PAL), a major enzyme involved in the phenolic biosynthesis, is regulated by light quality [74]. Far-red, red and blue wavelengths are especially the first step for increasing a precursor of the phenolic, shikimic acid [62]. In our experiment, *PAL1* and *PAL4* expression was enhanced under R/B light in rocket leaves. However, the differences between LED lights in the expression of these genes were not significant, apart from R/B vs. R light, where the induction was significant under R/B light. Induction of PAL activity was also observed in R/B light in comparison to other light treatments in red curly lettuces [75]. The slight, but statistically significant decrease in leaf phenol content under W light was not accompanied by a significant decrease in key genes. However, plant age is suggested to play a significant role in the transcriptome makeup: in mature lettuce plants cv. Batavia, R/B light induced a more distinctive transcriptome response in comparison to R/B light exposure of younger plants, and genes involved in the flavonoid biosynthesis were more upregulated in mature plants [76]. Furthermore, in our experiment, genes encoding for proteins-producing hydroxycinnamic acids were more expressed in the RNA-seq dataset of rocket leaves than the genes encoding for proteins that produce flavonols, in agreement with the previous results, where total phenolic content in the leaves of *B. rapa* mostly consisted of hydroxycinnamic acids, rather than flavonols [33]. It seems that the same applied to rocket leaves under any LED lights. Flavonoid and phenylpropanoid biosynthesis were two KEGG pathways significantly enriched with DEGs in W vs. B (eight genes downregulated by W light) and W vs. G (15 genes upregulated/eight genes downregulated) comparisons, respectively, in potato plantlets growing under different LED lights [77]. 

The production and accumulation of biochemical compounds have a direct effect on the nutritive and economic value of leafy vegetables [78]. The measurement of total soluble solids is a valuable indicator of tastiness, which greatly determines consumer preferences. Similarly, lamb lettuce exhibited a higher sugar content under the treatment comprising 90% red/10% blue wavelengths compared to other red-blue treatments [79], proving the necessity of red light in relatively high portions for the accumulation of sugars. *Brassica* vegetables contain several types of soluble sugars, such as sucrose and its derivatives, glucose and fructose. At the soluble solid level, LED lighting had a direct and clear effect on the Brix content of rocket leaves. R light positively affected fruit total soluble solid content, agreeing with previous results where an increased ratio of R light to G and B, followed by a 12/12 h photoperiod, caused an increase in sugar content [20]. Further exploring the molecular mechanisms behind this increase in sugar content, we report that this was accompanied by an upregulation of specific and crucial genes, such as *SUS1* and *CWINV1* homologs. SUS enzymes catalyze the reversible hydrolysis of sucrose to fructose and UDP-glucose, while INVs are responsible for the irreversible hydrolysis of sucrose to fructose and glucose [80]. Arabidopsis has six *SUS* isoforms, six cell wall acid invertases (*CWINVs*), two vacuole invertase (*INV*) genes and nine neutral/alkaline invertases (*INVs*). We have found several *SUS* genes expressed in our transcriptome dataset, but only *SUS1* exhibited a significant differential expression between different LED light treatments. In *B. rapa* ssp. *campestris*, it was found that the upregulation of *SUS1b*, *SUS3* and *SUS5*, caused by the silencing of two copies of an *BrOG1* gene, caused a reduction in fructose, glucose and total SSC, while *INV* activity remained unaltered [81]. The enhanced expression of *SUS1* under R light could indicate the rapid hydrolysis of sucrose to fructose and UDP-glucose in our experiment, and the enhancement of total SSC comprising these two types of sugars, since glucose and fructose are the main sugars found in different rocket accessions [82]. These data agree with data from tomato, where it was recently shown that far-red lighting coupled with R/B increases tomato fruit sugars at 30 DAA, by upregulating *SUS1*, *SUS3*, and invertases *LIN5* and *LIN7* genes that are important in fruit sugar content, sugar transportation and metabolism [83]. R light enhanced SSC in grape plantlets growing in vitro in comparison to W, B and Green (G) light, which was accompanied by a significantly enriched sucrose metabolism pathway with DEGs, where genes related to glycosyl hydrolase and hexosyl transferase were upregulated [12]. A study by [84] found that supplemental interlighting, comprising a mix of R and B light (R:B = 3), placed within the tomato plant canopy (on the side of hydroponically growing greenhouse plants), did not affect soluble solid content, agreeing with previous results [85]. Recently, a study using tomato germplasm showed that cultivars that have a higher brix content under LED light conditions could be developed through breeding. Using genes that might be implicated as markers, such as *SUS1*, could facilitate this process. As agriculture is moving towards modern and alternative growing systems with supplemental lighting, our results pave the way for the use of specific transcriptomic knowledge for plant breeding of vegetables such as rocket, where sugar concentration is a major determinant of taste and consumer acceptance. 

Apart from nutritive characteristics, an important aspect of plant production is product safety. Leafy vegetables are known to accumulate large amounts of nitrate ions, which are a source of carcinogenic compounds called nitrosamines [86]. Rocket is the single greatest nitrate accumulator among leafy vegetables. The above led the European Commission to impose a regulation (No 1258/2011) allowing a maximum of 6000 or 7000 mg NO_3_/kg of rocket fresh weight on harvest (April–September and October–March, respectively). For comparison, the maximally allowed nitrate amounts in lettuce and spinach are 3000–5000 and 3500 mg NO_3_/kg of fresh weight, respectively. Therefore, rocket cultivation should also focus on methods leading to nitrate reduction. In the present study, nitrate amounts were within the limits allowed by the EU and considerably lower under the red and blue containing LED treatments (R, R/B, and B) compared to W, but no differences were exhibited among LEDs. In a relevant study in *D. tenuifolia*, it was found that R light decreases the leaf nitrate content in comparison to B light; however, this decrease was further controlled by the level of nitrogen in the nutrient solution [21]. A lower nitrate content was reported under increasing red wavelength portion in green and red leaf lettuce [87], as well as in various stages of lettuce growth, from sprouts and microgreens to baby leaves [88]. Our results could be attributed to the activity of the enzyme nitrate reductase, as also stated by [89]. As our RNA-seq analysis results show, the nitrate reductase gene was induced under R light compared to all other lights. Additionally, there were four transcripts characterized as nitrate reductase in our rocket dataset, all which were significantly enhanced under R light in comparison to all other lights (data not shown). Enhanced gene transcription could lead to a higher production of the nitrate reductase enzyme, that explains the lower nitrate content under R light. Induced NR activity under R LED light has been reported in the past and it is known that R LED light decreases nitrate concentration in plants [89], and even more specifically in rocket *D. tenuifolia*, where it is suggested that light treatment can tailor nitrate concentrations in indoor cultivation [21]. 

Finally, in this study, yield expressed in fresh weight was slightly lesser in LED lights than the control W light, with R light producing the least, while no statistically significant differences were observed between the light treatments. This is an intriguing finding that could be attributed to the high variability that is often observed in rocket *D. tenuifolia* genetic material. This certainly imposes limitations, such as no concrete trend or that evidence can be obtained on the impact of LED lights on yield under these circumstances. More studies are needed with genetic material that will possibly exhibit less genetic variability, in order to get a clear picture on the yield trends of light treatments. After all, it is well known that results of the impact on LED lights change vastly between species and even within them [90]. However, our results show that LED lights can be used to improve quality characteristics, such as total phenolic content, SSC and lower nitrate concentrations, if the aim is to produce enough yield with a higher quality. More importantly, this study can be used as a concept study that shows vividly how genomics can be successfully combined with phenotypic approaches in order to tailor indoor plant production by deeply exploring the guiding molecular mechanisms that regulate and govern the expression of desirable phenotypes [91]. The elucidation of the role of genes that participate in the manifestation of important plant characteristics gives the opportunity for interference in the form of manipulation of these genes for the improvement of yield and quality of crops grown under these new growing regimes. 

## 5. Conclusions

In summary, this study is a comprehensive exploration of the effects of different LED lights on rocket growing under protected cultivation, in a controlled-environment cultivation system. By applying three different LED light treatments, designated as B, R/B, R, and W light as the control, we examined the effect on plant phenotype (yield and quality), as well as the plant physiological and biochemical status. Using RNA-seq technology, we went deeper into the leaf transcriptome to elucidate the molecular mechanisms that underline important procedures affected by light, such as photosynthesis and phenylpropanoids. With the horticultural LED lighting systems gaining popularity in controlled indoor cultivation systems, it is necessary to understand how using LED technology can achieve the best yield and quality of production, along with optimal resource-use efficiency. Further studies are needed to define how other light characteristics, such as light intensity, quality and photoperiod, influence gene expression of rocket cultivated in indoor growing systems. Extensive screening of rocket genetic material using modern molecular and analytical tools is also necessary to spot which cultivars could be more easily adapted and perform better in terms of yield and quality under these new growing systems.

## Figures and Tables

**Table 1:** Technical specifications according to the manufacturer. The percentage distribution for the defined wavelength ranges were measured according to a hand-held spectrometer.

LED Treatment		R:B = 0.3 (B)	R:B = 1 (R/B)	R:B = 4 (R)	W
Manufacturer		Slim-Spec Leafy Vegetables, Grow-Spec, (Zabrze, POLAND)	Slim-Spec Germination, Grow-Spec, (Zabrze, POLAND)	Slim-Spec Flower, Grow-Spec, (Zabrze, POLAND)	VT-1249, V-TAC, (Plovdiv, BULGARIA)
PPF (μmol s^−1^)		106	111.3	145.2	N/A
Par efficacy (μmol J^−1^)		2	2.1	2.2	N/A
Rated Power (W)		40	40	50	36
PPFD (μmol m^−2^ s^−1^)	Blue (400–500 nm)	75.3	80.6	26.8	35.4
	Green (500–600 nm)	39.3	17.9	24.8	95.6
	Red (600–700 nm)	84.8	102.6	149.7	69.5
	Far Red (700–780 nm)	18.4	2.1	42.8	7.2
λp (nm) Peak Wavelength		451	449	667	450

N/A: Not available data.

**Table 2:** Fresh weight (in g m^−2^), water-use efficiency (WUE, in g Fw L^−1^ H_2_O), energy-use efficiency (EUE, in (g FW kWh^−1^)), and land-use efficiency in one and four layers (LUE, kg m^−2^ a^−1^), and their standard errors. Same letters denote no statistically significant differences.

	Fresh Weight	Water-Use Efficiency	Energy-Use Efficiency	Land-Use Efficiency	
				1 Layer	4 Layers
R	679a ± 59.91	4.59a ± 0.40	5.54a ± 0.49	9.50a ± 0.84	38.00a ± 3.36
W	1113a ± 243.08	7.78a ± 1.7	11.79a ± 2.58	15.58a ± 3.40	62.31a ± 13.61
R/B	861a ± 180.14	5.28a ± 1.11	8.41a ± 1.76	12.06a ± 2.52	48.22a ± 10.09
B	997a ± 380.53	7.22a ± 2.76	9.74a ± 3.72	13.96a ± 5.33	55.83a ± 21.31

## Data Availability

Data available on request due to restrictions. The data presented in this study are available on request from the corresponding author. The data are not publicly available due to privacy restrictions.

## Supplementary Materials

The following supporting information can be downloaded at: https://www.mdpi.com/article/10.3390/plants12061203/s1, Figure S1: Water use efficiency (WUE) (A), energy use efficiency (EUE) (B) and land use efficiencies (LUE) for rocket plants growing under different LED lights R, B, R/B and under W light. Same letters denote non statistically significant differences at *p* ≤ 0.05. Figure S2: Expression for four rocket gene transcripts using RNA-seq data analysis (FPKM values – first left y axis, orange columns) and experimental RT-PCR analysis (ΔΔCt – second right y axis, blue lines). Spreadsheet S1.

## References

[1] Pasini F., Verardo V., Cerretani L., Caboni M.F., D’Antuono L.F. (2011). Rocket Salad (*Diplotaxis* and *Eruca* spp.) Sensory Analysis and Relation with Glucosinolate and Phenolic Content. J. Sci. Food Agric..

[2] Caruso G., Parrella G., Giorgini M., Nicoletti R. (2018). Crop Systems, Quality and Protection of *Diplotaxis tenuifolia*. Agriculture.

[3] Bozokalfa K.M., Eşiyok D., Yağmur B. (2011). Use of Multivariate Analysis in Mineral Accumulation of Rocket (*Eruca sativa*) Accessions. Genetika.

[4] Pieroni A., Quave C.L., Santoro R.F. (2004). Folk Pharmaceutical Knowledge in the Territory of the Dolomiti Lucane, Inland Southern Italy. J. Ethnopharmacol..

[5] Chen M., Chory J., Fankhauser C. (2004). Light Signal Transduction in Higher Plants. Annu. Rev. Genet..

[6] Miao Y., Wang X., Gao L., Chen Q., Mei Q.U. (2016). Blue Light Is More Essential than Red Light for Maintaining the Activities of Photosystem II and I and Photosynthetic Electron Transport Capacity in Cucumber Leaves. J. Integr. Agric..

[7] Christiaens A., Gobin B., van Labeke M.-C. Light Quality and Adventitious Rooting: A Mini-Review. Proceedings of the VIII International Symposium on Light in Horticulture 1134.

[8] Langhans R.W., Tibbitts T.W. (1997). Plant Growth Chamber Handbook.

[9] Kozuka T., Suetsugu N., Wada M., Nagatani A. (2013). Antagonistic Regulation of Leaf Flattening by Phytochrome B and Phototropin in *Arabidopsis thaliana*. Plant Cell Physiol..

[10] De Carbonnel M., Davis P., Roelfsema M.R.G., Inoue S., Schepens I., Lariguet P., Geisler M., Shimazaki K., Hangarter R., Fankhauser C. (2010). The Arabidopsis Phytochrome Kinase Substrate2 Protein Is a Phototropin Signaling Element That Regulates Leaf Flattening and Leaf Positioning. Plant Physiol..

[11] Bantis F., Smirnakou S., Ouzounis T., Koukounaras A., Ntagkas N., Radoglou K. (2018). Current Status and Recent Achievements in the Field of Horticulture with the Use of Light-Emitting Diodes (LEDs). Sci. Hortic..

[12] Li C.-X., Xu Z.-G., Dong R.-Q., Chang S.-X., Wang L.-Z., Khalil-Ur-Rehman M., Tao J.-M. (2017). An RNA-Seq Analysis of Grape Plantlets Grown In Vitro Reveals Different Responses to Blue, Green, Red LED Light, and White Fluorescent Light. Front. Plant Sci..

[13] Deng Y., Yao J., Wang X., Guo H., Duan D. (2012). Transcriptome Sequencing and Comparative Analysis of *Saccharina japonica* (Laminariales, Phaeophyceae) under Blue Light Induction. PLoS ONE.

[14] Liu X., Chen Z., Jahan M.S., Wen Y., Yao X., Ding H., Guo S., Xu Z. (2020). RNA-Seq Analysis Reveals the Growth and Photosynthetic Responses of Rapeseed *(Brassica napus* L.) under Red and Blue LEDs with Supplemental Yellow, Green, or White Light. Hortic. Res..

[15] Piovene C., Orsini F., Bosi S., Sanoubar R., Bregola V., Dinelli G., Gianquinto G. (2015). Optimal Red: Blue Ratio in Led Lighting for Nutraceutical Indoor Horticulture. Sci. Hortic..

[16] Pennisi G., Blasioli S., Cellini A., Maia L., Crepaldi A., Braschi I., Spinelli F., Nicola S., Fernandez J.A., Stanghellini C. (2019). Unraveling the Role of Red:Blue LED Lights on Resource Use Efficiency and Nutritional Properties of Indoor Grown Sweet Basil. Front. Plant Sci..

[17] Pennisi G., Orsini F., Blasioli S., Cellini A., Crepaldi A., Braschi I., Spinelli F., Nicola S., Fernandez J.A., Stanghellini C. (2019). Resource Use Efficiency of Indoor Lettuce (*Lactuca Sativa* L.) Cultivation as Affected by Red: Blue Ratio Provided by LED Lighting. Sci. Rep..

[18] Zhang Y., Kaiser E., Zhang Y., Yang Q., Li T. (2019). Red/Blue Light Ratio Strongly Affects Steady-state Photosynthesis, but Hardly Affects Photosynthetic Induction in Tomato (*Solanum lycopersicum*). Physiol. Plant.

[19] Metallo R.M., Kopsell D.A., Sams C.E., Bumgarner N.R. (2018). Influence of Blue/Red vs. White LED Light Treatments on Biomass, Shoot Morphology, and Quality Parameters of Hydroponically Grown Kale. Sci. Hortic..

[20] Elmardy N.A., Yousef A.F., Lin K., Zhang X., Ali M.M., Lamlom S.F., Kalaji H.M., Kowalczyk K., Xu Y. (2021). Photosynthetic Performance of Rocket (*Eruca sativa*. Mill.) Grown under Different Regimes of Light Intensity, Quality, and Photoperiod. PLoS ONE.

[21] Signore A., Bell L., Santamaria P., Wagstaff C., van Labeke M.-C. (2020). Red Light Is Effective in Reducing Nitrate Concentration in Rocket by Increasing Nitrate Reductase Activity, and Contributes to Increased Total Glucosinolates Content. Front. Plant Sci..

[22] Cavaiuolo M., Cocetta G., Spadafora N.D., Müller C.T., Rogers H.J., Ferrante A. (2017). Gene Expression Analysis of Rocket Salad under Pre-Harvest and Postharvest Stresses: A Transcriptomic Resource for *Diplotaxis tenuifolia*. PLoS ONE.

[23] Danner M., Locherer M., Hank T., Richter K., Consortium E. (2015). Measuring Leaf Area Index (LAI) with the LI-Cor LAI 2200C or LAI-2200 (+2200Clear Kit)—Theory, Measurement, Problems, Interpretation. https://www.researchgate.net/publication/337784279_Measuring_Leaf_Area_Index_LAI_with_the_LI-Cor_LAI_2200C_or_LAI-2200_2200Clear_Kit_EnMAP_Field_Guides_Technical_Report.

[24] Singleton V.L., Rossi J.A. (1965). Colorimetry of Total Phenolics with Phosphomolybdic-Phosphotungstic Acid Reagents. Am. J. Enol. Vitic..

[25] Benzie I.F.F., Strain J.J. (1996). The Ferric Reducing Ability of Plasma (FRAP) as a Measure of “Antioxidant Power”: The FRAP Assay. Anal. Biochem..

[26] Cataldo D.A., Maroon M., Schrader L.E., Youngs V.L. (1975). Rapid Colorimetric Determination of Nitrate in Plant Tissue by Nitration of Salicylic Acid. Commun. Soil Sci. Plant Anal..

[27] Moustaka J., Tanou G., Giannakoula A., Adamakis I.-D.S., Panteris E., Eleftheriou E.P., Moustakas M. (2020). Anthocyanin Accumulation in Poinsettia Leaves and Its Functional Role in Photo-Oxidative Stress. Environ. Exp. Bot..

[28] Bilger W., Schreiber U., Bock M. (1995). Determination of the Quantum Efficiency of Photosystem II and of Non-Photochemical Quenching of Chlorophyll Fluorescence in the Field. Oecologia.

[29] Grabherr M.G., Haas B.J., Yassour M., Levin J.Z., Thompson D.A., Amit I., Adiconis X., Fan L., Raychowdhury R., Zeng Q. (2011). Trinity: Reconstructing a Full-Length Transcriptome without a Genome from RNA-Seq Data. Nat. Biotechnol..

[30] Love M.I., Huber W., Anders S. (2014). Moderated Estimation of Fold Change and Dispersion for RNA-Seq Data with DESeq2. Genome Biol..

[31] Young M.D., Wakefield M.J., Smyth G.K., Oshlack A. (2010). Gene Ontology Analysis for RNA-Seq: Accounting for Selection Bias. Genome Biol..

[32] Chen C., Chen H., Zhang Y., Thomas H.R., Frank M.H., He Y., Xia R. (2020). TBtools: An Integrative Toolkit Developed for Interactive Analyses of Big Biological Data. Mol. Plant.

[33] Francisco M., Ali M., Ferreres F., Moreno D.A., Velasco P., Soengas P. (2016). Organ-Specific Quantitative Genetics and Candidate Genes of Phenylpropanoid Metabolism in Brassica Oleracea. Front. Plant Sci..

[34] Kramer D.M., Johnson G., Kiirats O., Edwards G.E. (2004). New Fluorescence Parameters for the Determination of QA Redox State and Excitation Energy Fluxes. Photosynth. Res..

[35] Paik I., Huq E. (2019). Plant Photoreceptors: Multi-Functional Sensory Proteins and Their Signaling Networks. Semin. Cell Dev. Biol..

[36] Mohidul Hasan M., Bashir T., Ghosh R., Lee S.K., Bae H. (2017). An Overview of LEDs’ Effects on the Production of Bioactive Compounds and Crop Quality. Molecules.

[37] Choi H.G., Moon B.Y., Kang N.J. (2015). Effects of LED Light on the Production of Strawberry during Cultivation in a Plastic Greenhouse and in a Growth Chamber. Sci. Hortic..

[38] Yorio N.C., Goins G.D., Kagie H.R., Wheeler R.M., Sager J.C. (2001). Improving Spinach, Radish, and Lettuce Growth under Red Light-Emitting Diodes (LEDs) with Blue Light Supplementation. HortScience.

[39] Chen X., Guo W., Xue X., Wang L., Qiao X. (2014). Growth and Quality Responses of ‘Green Oak Leaf’Lettuce as Affected by Monochromic or Mixed Radiation Provided by Fluorescent Lamp (FL) and Light-Emitting Diode (LED). Sci. Hortic..

[40] Alrajhi A.A., Alsahli A.S., Alhelal I.M., Rihan H.Z., Fuller M.P., Alsadon A.A., Ibrahim A.A. (2023). The Effect of LED Light Spectra on the Growth, Yield and Nutritional Value of Red and Green Lettuce (*Lactuca Sativa*). Plants.

[41] Chang C.-L., Chang K.-P. (2014). The Growth Response of Leaf Lettuce at Different Stages to Multiple Wavelength-Band Light-Emitting Diode Lighting. Sci. Hortic..

[42] Naznin M.T., Lefsrud M., Gravel V., Azad M.O.K. (2019). Blue Light Added with Red LEDs Enhance Growth Characteristics, Pigments Content, and Antioxidant Capacity in Lettuce, Spinach, Kale, Basil, and Sweet Pepper in a Controlled Environment. Plants.

[43] Zhang X., Bian Z., Yuan X., Chen X., Lu C. (2020). A Review on the Effects of Light-Emitting Diode (LED) Light on the Nutrients of Sprouts and Microgreens. Trends Food Sci. Technol..

[44] Viršilė A., Miliauskienė J., Haimi P.J., Laužikė K., Samuolienė G. (2020). The Comparison of Constant and Dynamic Red and Blue Light Irradiation Effects on Red and Green Leaf Lettuce. Agronomy.

[45] Bantis F., Ouzounis T., Radoglou K. (2016). Artificial LED Lighting Enhances Growth Characteristics and Total Phenolic Content of *Ocimum basilicum*, but Variably Affects Transplant Success. Sci. Hortic..

[46] Pashkovskiy P.P., Soshinkova T.N., van Korolkova D., van Kartashov A., Zlobin I.E., Lyubimov V.Y., Kreslavski V.D., van Kuznetsov V. (2018). The Effect of Light Quality on the Pro-/Antioxidant Balance, Activity of Photosystem II, and Expression of Light-Dependent Genes in Eutrema Salsugineum Callus Cells. Photosynth. Res..

[47] Gawroński P., Witoń D., Vashutina K., Bederska M., Betliński B., Rusaczonek A., Karpiński S. (2014). Mitogen-Activated Protein Kinase 4 Is a Salicylic Acid-Independent Regulator of Growth but Not of Photosynthesis in Arabidopsis. Mol. Plant.

[48] Moustakas M., Moustaka J., Sperdouli I. (2022). Hormesis in Photosystem II: A Mechanistic Understanding. Curr. Opin. Toxicol..

[49] Krieger-Liszkay A. (2005). Singlet Oxygen Production in Photosynthesis. J. Exp. Bot..

[50] Kasajima I., Takahara K., Kawai-Yamada M., Uchimiya H. (2009). Estimation of the Relative Sizes of Rate Constants for Chlorophyll De-Excitation Processes through Comparison of Inverse Fluorescence Intensities. Plant Cell Physiol..

[51] Adamakis I.-D.S., Sperdouli I., Eleftheriou E.P., Moustakas M. (2020). Hydrogen Peroxide Production by the Spot-like Mode Action of Bisphenol A. Front. Plant Sci..

[52] Liu Y., Wang T., Fang S., Zhou M., Qin J. (2018). Responses of Morphology, Gas Exchange, Photochemical Activity of Photosystem II, and Antioxidant Balance in *Cyclocarya paliurus* to Light Spectra. Front. Plant Sci..

[53] Bian Z., Yang Q., Li T., Cheng R., Barnett Y., Lu C. (2018). Study of the Beneficial Effects of Green Light on Lettuce Grown under Short-term Continuous Red and Blue Light-emitting Diodes. Physiol. Plant.

[54] Sperdouli I., Moustakas M. (2014). A Better Energy Allocation of Absorbed Light in Photosystem II and Less Photooxidative Damage Contribute to Acclimation of Arabidopsis Thaliana Young Leaves to Water Deficit. J. Plant Physiol..

[55] Serôdio J., Campbell D.A. (2021). Photoinhibition in Optically Thick Samples: Effects of Light Attenuation on Chlorophyll Fluorescence-Based Parameters. J. Theor. Biol..

[56] Nickelsen J., Rengstl B. (2013). Photosystem II Assembly: From Cyanobacteria to Plants. Annu. Rev. Plant Biol..

[57] Xingxing C., Jiuyang L., Huan Z., Fudong L., Shuya Z., Min X., Ke R., Yuhua W., Aigen F. (2018). Crystal Structure of Psb27 from Arabidopsis Thaliana Determined at a Resolution of 1.85 Å. Photosynth. Res..

[58] Dietzel L., Bräutigam K., Steiner S., Schüffler K., Lepetit B., Grimm B., Schöttler M.A., Pfannschmidt T. (2011). Photosystem II Supercomplex Remodeling Serves as an Entry Mechanism for State Transitions in Arabidopsis. Plant Cell.

[59] Hou X., Fu A., Garcia V.J., Buchanan B.B., Luan S. (2015). PSB27: A Thylakoid Protein Enabling Arabidopsis to Adapt to Changing Light Intensity. Proc. Natl. Acad. Sci. USA.

[60] Tian Y., Wang H., Zhang Z., Zhao X., Wang Y., Zhang L. (2022). An RNA-Seq Analysis Reveals Differential Transcriptional Responses to Different Light Qualities in Leaf Color of *Camellia sinensis* Cv. Huangjinya. J. Plant Growth Regul..

[61] Chotewutmontri P., Barkan A. (2020). Light-Induced PsbA Translation in Plants Is Triggered by Photosystem II Damage via an Assembly-Linked Autoregulatory Circuit. Proc. Natl. Acad. Sci. USA.

[62] Huo Y., Wang M., Wei Y., Xia Z. (2016). Overexpression of the Maize PsbA Gene Enhances Drought Tolerance through Regulating Antioxidant System, Photosynthetic Capability, and Stress Defense Gene Expression in Tobacco. Front. Plant Sci..

[63] Wei L., Guo J., Ouyang M., Sun X., Ma J., Chi W., Lu C., Zhang L. (2010). LPA19, a Psb27 Homolog in Arabidopsis Thaliana, Facilitates D1 Protein Precursor Processing during PSII Biogenesis. J. Biol. Chem..

[64] Chen H., Zhang D., Guo J., Wu H., Jin M., Lu Q., Lu C., Zhang L. (2006). A Psb27 Homologue in Arabidopsis Thaliana Is Required for Efficient Repair of Photodamaged Photosystem II. Plant Mol. Biol..

[65] Plöchinger M., Schwenkert S., von Sydow L., Schröder W.P., Meurer J. (2016). Functional Update of the Auxiliary Proteins PsbW, PsbY, HCF136, PsbN, TerC and ALB3 in Maintenance and Assembly of PSII. Front. Plant Sci..

[66] Yousef A.F., Ali M.M., Rizwan H.M., Tadda S.A., Kalaji H.M., Yang H., Ahmed M.A.A., Wróbel J., Xu Y., Chen F. (2021). Photosynthetic Apparatus Performance of Tomato Seedlings Grown under Various Combinations of LED Illumination. PLoS ONE.

[67] Yu W., Liu Y., Song L., Jacobs D.F., Du X., Ying Y., Shao Q., Wu J. (2017). Effect of Differential Light Quality on Morphology, Photosynthesis, and Antioxidant Enzyme Activity in Camptotheca Acuminata Seedlings. J. Plant Growth Regul..

[68] Yamori W. (2016). Photosynthetic Response to Fluctuating Environments and Photoprotective Strategies under Abiotic Stress. J. Plant Res..

[69] Ostan R., Lanzarini C., Pini E., Scurti M., Vianello D., Bertarelli C., Fabbri C., Izzi M., Palmas G., Biondi F. (2015). Inflammaging and Cancer: A Challenge for the Mediterranean Diet. Nutrients.

[70] Lattanzio V., Lattanzio V.M.T., Cardinali A. (2006). Role of Phenolics in the Resistance Mechanisms of Plants against Fungal Pathogens and Insects. Phytochem. Adv. Res..

[71] Kopsell D.A., Kopsell D.E., Lefsrud M.G., Curran-Celentano J., Dukach L.E. (2004). Variation in Lutein, β-Carotene, and Chlorophyll Concentrations among Brassica Oleracea Cultigens and Seasons. HortScience.

[72] Bantis F. (2021). Light Spectrum Differentially Affects the Yield and Phytochemical Content of Microgreen Vegetables in a Plant Factory. Plants.

[73] Giménez A., Martínez-Ballesta M.d.C., Egea-Gilabert C., Gómez P.A., Artés-Hernández F., Pennisi G., Orsini F., Crepaldi A., Fernández J.A. (2021). Combined Effect of Salinity and LED Lights on the Yield and Quality of Purslane (*Portulaca oleracea* L.) Microgreens. Horticulturae.

[74] Lister C.E., Lancaster J.E., Walker J.R.L. (1996). Phenylalanine Ammonia-Lyase (PAL) Activity and Its Relationship to Anthocyanin and Flavonoid Levels in New Zealand-Grown Apple Cultivars. J. Am. Soc. Hortic. Sci..

[75] Heo J.-W., Kang D.-H., Bang H.-S., Hong S.-G., Chun C.-H., Kang K.-K. (2012). Early Growth, Pigmentation, Protein Content, and Phenylalanine Ammonia-Lyase Activity of Red Curled Lettuces Grown under Different Lighting Conditions. Hortic. Sci. Technol..

[76] Sng B.J.R., Mun B., Mohanty B., Kim M., Phua Z.W., Yang H., Lee D.-Y., Jang I.-C. (2021). Combination of Red and Blue Light Induces Anthocyanin and Other Secondary Metabolite Biosynthesis Pathways in an Age-Dependent Manner in Batavia Lettuce. Plant Sci..

[77] Chen L., Yang Y., Jiang Y., Zhao J., Zang H., Wang X., Hu Y., Xue X. (2019). RNA-Seq Analysis Reveals Differential Responses of Potato (*Solanum tuberosum* L.) Plantlets Cultured in Vitro to Red, Blue, Green, and White Light-Emitting Diodes (LEDs). J. Plant Growth Regul..

[78] Lin K.-H., Huang M.-Y., Huang W.-D., Hsu M.-H., Yang Z.-W., Yang C.-M. (2013). The Effects of Red, Blue, and White Light-Emitting Diodes on the Growth, Development, and Edible Quality of Hydroponically Grown Lettuce (*Lactuca sativa* L. var. *capitata*). Sci. Hortic..

[79] Wojciechowska R., Długosz-Grochowska O., Kołton A., Żupnik M. (2015). Effects of LED Supplemental Lighting on Yield and Some Quality Parameters of Lamb’s Lettuce Grown in Two Winter Cycles. Sci. Hortic..

[80] Stein O., Granot D. (2019). An Overview of Sucrose Synthases in Plants. Front. Plant Sci..

[81] Jiang M., Zhan Z., Li H., Dong X., Cheng F., Piao Z. (2020). *Brassica Rapa* Orphan Genes Largely Affect Soluble Sugar Metabolism. Hortic. Res..

[82] Bell L., Yahya H.N., Oloyede O.O., Methven L., Wagstaff C. (2017). Changes in Rocket Salad Phytochemicals within the Commercial Supply Chain: Glucosinolates, Isothiocyanates, Amino Acids and Bacterial Load Increase Significantly after Processing. Food Chem..

[83] Ji Y., Nunez Ocana D., Choe D., Larsen D.H., Marcelis L.F.M., Heuvelink E. (2020). Far-red Radiation Stimulates Dry Mass Partitioning to Fruits by Increasing Fruit Sink Strength in Tomato. New Phytol..

[84] Paucek I., Pennisi G., Pistillo A., Appolloni E., Crepaldi A., Calegari B., Spinelli F., Cellini A., Gabarrell X., Orsini F. (2020). Supplementary LED Interlighting Improves Yield and Precocity of Greenhouse Tomatoes in the Mediterranean. Agronomy.

[85] Dzakovich M.P., Gómez C., Mitchell C.A. (2015). Tomatoes Grown with Light-Emitting Diodes or High-Pressure Sodium Supplemental Lights Have Similar Fruit-Quality Attributes. HortScience.

[86] Addiscott T. (2006). Is It Nitrate That Threatens Life or the Scare about Nitrate?. J. Sci. Food Agric..

[87] Viršilė A., Brazaitytė A., Vaštakaitė-Kairienė V., Jankauskienė J., Miliauskienė J., Samuolienė G., Novičkovas A., Duchovskis P. (2019). Nitrate, Nitrite, Protein, Amino Acid Contents, and Photosynthetic and Growth Characteristics of Tatsoi Cultivated under Various Photon Flux Densities and Spectral Light Compositions. Sci. Hortic..

[88] Ferrón-Carrillo F., Guil-Guerrero J.L., González-Fernández M.J., Lyashenko S., Battafarano F., da Cunha-Chiamolera T.P.L., Urrestarazu M. (2021). LED Enhances Plant Performance and Both Carotenoids and Nitrates Profiles in Lettuce. Plant Foods Hum. Nutr..

[89] Lillo C., Appenroth K.-J. (2001). Light Regulation of Nitrate Reductase in Higher Plants: Which Photoreceptors Are Involved?. Plant Biol..

[90] Al Murad M., Razi K., Jeong B.R., Samy P.M.A., Muneer S. (2021). Light Emitting Diodes (LEDs) as Agricultural Lighting: Impact and Its Potential on Improving Physiology, Flowering, and Secondary Metabolites of Crops. Sustainability.

[91] Marondedze C., Liu X., Huang S., Wong C., Zhou X., Pan X., An H., Xu N., Tian X., Wong A. (2018). Towards a Tailored Indoor Horticulture: A Functional Genomics Guided Phenotypic Approach. Hortic. Res..

