# Supplementary material for: LED omics in Rocket Salad (Diplotaxis tenuifolia): Comparative Analysis in Different Light-Emitting Diode (LED) Spectrum and Energy Consumption
Source: Plants (Basel). 2023 Mar 7;12(6):1203. doi: 10.3390/plants12061203 (PMC10059670; doi:10.3390/plants12061203)
Supplement: Supplementary file 1 [file plants-12-01203-s001.zip › plants-2221840-supplementary.pdf]

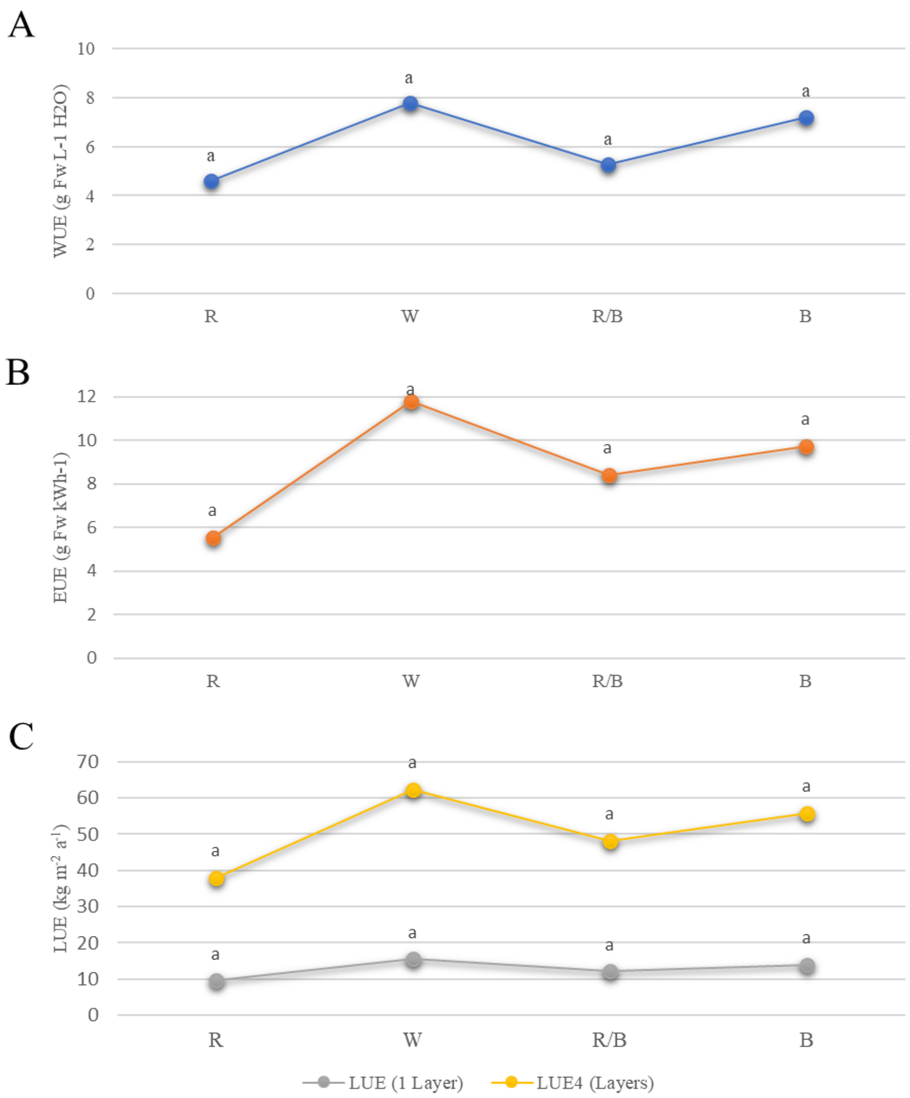

Figure S1. Water use efficiency (WUE) (A), energy use efficiency (EUE) (B) and land use efficiencies (LUE) for rocket plants growing under different LED lights R, B, R/B and under W light. Same letters denote non statistically significant differences at *p* ≤ 0.05.

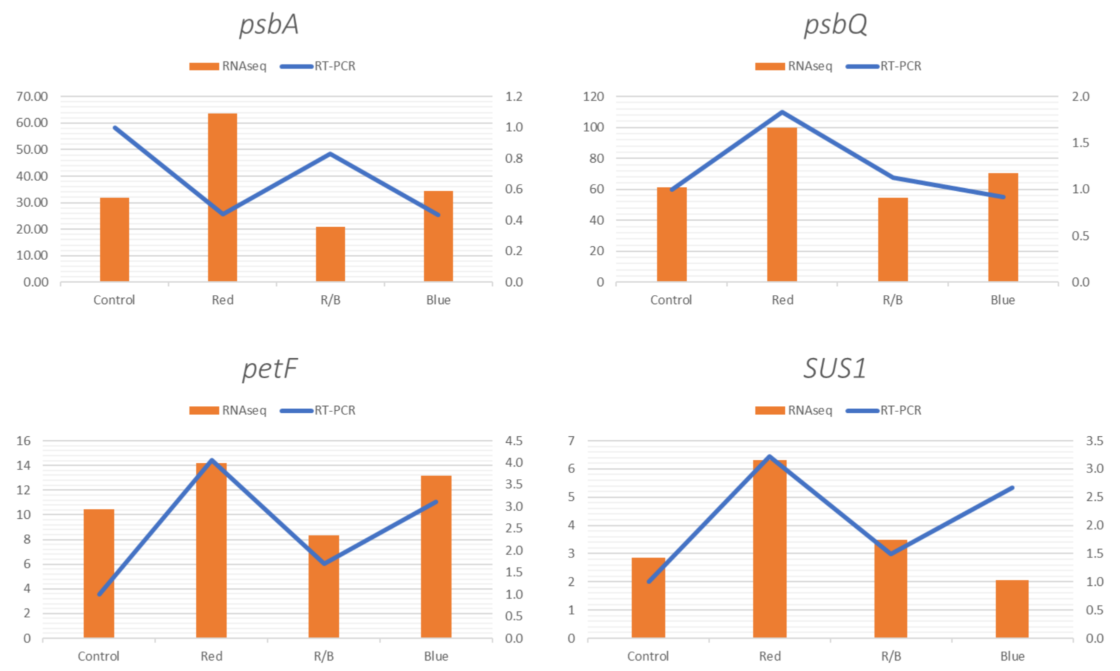

Figure S2. Expression for four rocket gene transcripts using RNA-seq data analysis (FPKM values – first left y axis, orange columns) and experimental RT-PCR analysis (ΔΔCt – second right y axis, blue lines).
